# Exploring the Therapeutic Potential of Nobiletin in Nonsmall Cell Lung Cancer

**DOI:** 10.1155/bmri/9477110

**Published:** 2026-06-26

**Authors:** Adam Haysom-McDowell, Jack Bartholomew, Stewart Yeung, Keshav Raj Paudel, Nenad Naumovski, Dennis Chang, Hui Chen, Parteek Prasher, Brian Gregory George Oliver, Frederick Williams, Kamal Dua, Gabriele De Rubis

**Affiliations:** ^1^ Discipline of Pharmacy, Graduate School of Health, University of Technology Sydney, Ultimo, New South Wales, Australia, uts.edu.au; ^2^ Woolcock Institute of Medical Research, Macquarie University, Sydney, New South Wales, Australia, mq.edu.au; ^3^ NICM Health Research Institute and School of Science, Western Sydney University, Westmead, New South Wales, Australia, westernsydney.edu.au; ^4^ Food, Chemical and Biotechnology Cluster, Singapore Institute of Technology, Punggol, Singapore, singaporetech.edu.sg; ^5^ Department of Nutrition and Dietetics, School of Health Science and Education, Harokopio University, Athens, Greece, hua.gr; ^6^ University of Canberra Research Institute for Sport and Exercise (UCRISE), University of Canberra, Canberra, Australian Capital Territory, Australia, canberra.edu.au; ^7^ School of Life Science, University of Technology Sydney, Ultimo, New South Wales, Australia, uts.edu.au; ^8^ Department of Chemistry, University of Petroleum and Energy Studies, Dehradun, India, upes.ac.in; ^9^ Department of Pharmacology and Experimental Therapeutics, University of Toledo, Toledo, Ohio, USA, utoledo.edu

**Keywords:** nobiletin, nonsmall cell lung cancer, phytoceutical, pulmonary, therapeutic

## Abstract

Nonsmall cell lung cancer (NSCLC) is one of the most prevalent cancers and leading causes of cancer‐related mortality worldwide. Despite advancements in medical treatment, current therapeutic strategies are often hindered by late‐stage diagnosis, high recurrence rates and resistance to therapies. These challenges highlight the urgent need for novel, less toxic and more effective treatment options. Phytoceuticals, bioactive plant‐derived compounds, have gained increasing attention for their potential to complement or enhance conventional cancer therapies. Among these, nobiletin, a polymethoxylated flavonoid found in citrus peels, has promising anti‐inflammatory, antioxidant and anticancer properties. This review explores the therapeutic potential of nobiletin in NSCLC, focusing on its ability to modulate key cancer hallmarks such as uncontrolled proliferation, metastasis, immune evasion and multidrug resistance. Preclinical studies suggest that nobiletin inhibits tumour growth, enhances chemosensitivity and suppresses metastasis through multiple pathways. However, its clinical application is currently limited by poor bioavailability, prompting the need for innovative delivery strategies. Therefore, this review also discusses emerging approaches to improve nobiletin′s biological activity, supporting further investigations into its role in NSCLC therapy.

## 1. Introduction

### 1.1. Lung Cancer

Lung cancer is a major contributor to the global public health burden. It is the most diagnosed cancer worldwide, accounting for 2.5 million new cases in 2022 (12.4% of all cases), followed by the breast (11.6%), colorectum (9.6%), prostate (7.3%) and stomach (4.9%) [1]. Despite recent advances in the treatment of lung cancer, it continues to be the leading cause of all cancer‐related deaths worldwide [1, 2]. Lung cancer is the fifth most diagnosed cancer in Australia (~15,100 cases in 2024) with just a 25% 5‐year survival [3].

**Figure 1:**
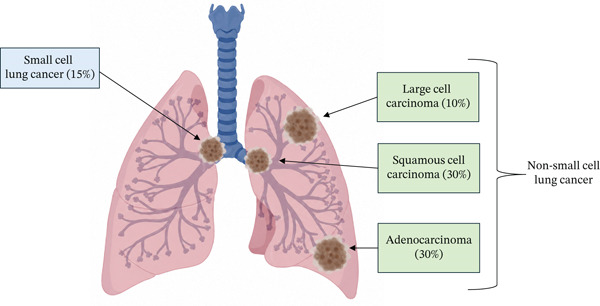
Localisation and distribution of various types of lung cancer is different regions of the lungs. Distribution of nonsmall cell lung cancer across different lung regions where adenocarcinoma (40%) and large cell carcinoma (10%) usually develop in the periphery of the lung compared with squamous cell carcinoma which usually develops in the mucous membrane of the central parts of the lung as depicted in the figure. The remaining 5% of NSCLC are of mixed or rare pathology (image created using https://BioRender.com).

Lung cancer can be categorised (Figure 1) as nonsmall cell lung cancer (NSCLC) (~85% of patients) or small cell lung cancer (SCLC) (~15%)[4] with the latter having a poorer prognosis [5]. The median survival duration for patients with SCLC is less than 2 years for patients diagnosed with early‐stage disease and less than 1 year for patients with metastatic disease. NSCLC can be subcategorised into adenocarcinoma (40% of all lung cancers), squamous‐cell carcinoma (25%) and large‐cell carcinoma (10%) [6, 7]. NSCLC has a more favourable prognosis due to its reduced metastatic characteristics compared with SCLC; however, its treatment is often marred by delayed diagnosis as the majority of cases (~70%) are detected at an advanced stage of disease [8].

Exposure to tobacco smoke is the most well‐established risk factor in lung carcinogenesis accounting for almost 85% of all lung cancer cases [4]. The remaining 15% of cases occur in nonsmokers and are of mixed or unknown aetiology [9]. Other risk factors include exposure to ionising radiation, radon and occupational hazards (e.g., asbestos exposure); older age and male sex ([4, 9], [10]). Recently, research has demonstrated the relationship between exposure to air pollution and lung carcinogenesis through an inflammatory axis [11]. Hill et al. have shown that just 3 years of exposure to particulate matter 2.5 (PM_2.5_) is sufficient to increase the risk of developing mutant epidermal growth factor receptor (EGFR) driven lung cancer [12].

Furthermore, a family history is known to be positively associated with the development of lung cancer, illustrating a component of familial genetic susceptibility. Several genetic mutations in key proteins such as EGFR, anaplastic lymphoma kinase (ALK) and c‐ros oncogene 1 (ROS1) have been proposed to influence disease development and may serve as targets for personalised treatment of NSCLC [13–15].

### 1.2. Current Therapeutic Options for the Treatment of NSCLC

The mainstay of NSCLC treatment includes surgery, radiotherapy, chemotherapy or molecularly targeted therapy. Generally, treatment options are guided by the stage of cancer progression, histological markers, genetic aberrations and the overall patient′s condition and preference(s) [16].

However, despite resection and a promising response to chemotherapy, up to 70% of patients will develop some form of chemoresistance, resulting in recurrence and disease progression [17, 18].

Stages IIIB and IIIC NSCLC refer to tumours that have metastasised to nearby mediastinal lymph nodes, or locally advanced tumours that may involve local lymph nodes. The management of Stage III NSCLC is complex and less well‐defined [19]. A combination of chemotherapy and radiation is the preferred treatment modality for these patients [20] with chemotherapy administered either concomitantly or sequentially with radiotherapy. Concomitant chemoradiotherapy has been shown to improve survival, with an absolute survival rate of 18.4% at 3 years and of 15.1% at 5 years [21]. The concurrent approach generally offers better survival outcomes, but it is characterised by increased risk of adverse effects such as esophagitis, haematological toxicity and pneumonitis [20].

Although difficult to quantify, the socioeconomic burden of NSCLC remains significant. A 2017 analysis of 191 patients in Italy found the cost to the Italian National Health Service per patient per year to be €18,921, with a mean treatment period of 16.4 months [22]. Another 2020 analysis of the Australian ‘45 and Up’ study found that the average cost to the healthcare system from 1 year prediagnosis to 3 years postdiagnosis of lung cancer was $51,000 per person [23]. The economic burden is further compounded by significant social consequences. Many NSCLC patients report that they are physically unable to work, which leads to a loss of income, social isolation and a reduced sense of purpose [24, 25]. The psychological implications from these social challenges commonly leads to the onset of depression and anxiety, and research indicates that cancer patients five times more likely to have depression compared with the general population [26]. A recent systematic review identified that 44% of people affected by cancer were negatively impacted by the psychosocial effects of cancer which, in turn, significantly reduced clinical and economic outcomes for patients and caregivers [27]. The poor clinical and social outcomes associated with traditional NSCLC therapies further demonstrate the need for novel approaches that are less costly, less toxic and less susceptible to treatment resistance.

### 1.3. Novel Therapies for NSCLC

In the past two decades, there has been significant advancement in the treatment of NSCLC with the development of molecular targeted therapy and immunotherapy, particularly in later stages of the disease [28].

Targeted cancer therapies interact with the aberrant genes and proteins that drive cancer development inhibiting the survival of cancer cells, whilst minimising damage to healthy, noncancerous tissues [29]. For example, EGFR tyrosine kinase inhibitors (TKIs) are effective treatment options for the 10%–50% of NSCLC patients with EGFR mutations [20]. The median progression‐free survival using osimertinib, a third‐generation EGFR‐TKI, after chemotherapy in Stage III EGFR‐mutated NSCLC was 39.1 months compared with 5.6 months in the placebo group [30]. However, even third‐generation EGFR‐TKIs have been shown to succumb to treatment resistance [28], and all patients will eventually experience disease progression [31]. Alternatively, immune checkpoint inhibitors such as nivolumab and pembrolizumab may restore T‐cell sensitivity by modulation of the programmed death‐ligand 1 (PD‐L1) pathway in tumour cells [15]. However, many patients with PD‐L1 mutations do not show any response to immune checkpoint inhibitors or develop resistance after 8 months of treatment [32].

The advancement of immunotherapy and molecular targeted therapy has improved the outlook for many patients living with NSCLC by offering a specific, yet less toxic treatment option alongside traditional treatment paradigms [28, 29]. No treatment, however, is without challenges. The novel molecular targeted therapy and immune checkpoint inhibitors continue to be undermined by treatment resistance [28, 29, 32], whereas targeted therapies are ineffective in patients that do not have a defined molecular abnormality, which constitutes the majority of lung cancer cases [33]. The heterogenous nature of NSCLC means that not all patients with a particular molecular abnormality will respond to treatment and, even when a molecular abnormality is identified, there are limited guidelines on the optimal treatment regime [29]. Finally, the cost associated with these agents can be prohibitive, and the resources required for advanced molecular testing further raise accessibility concerns [15, 29].

Despite promising advancements in the treatment of NSCLC, it continues to be a burdensome and complex disease with poor clinical outcomes [15]. In particular, the issue of resistance continues to undermine novel therapies and highlights the need for innovative, more accessible and holistic compounds that can target multiple mechanisms to reduce NSCLC treatment resistance and improve patient outcomes [29]. *In recent years, increasing attention has been given to phytoceuticals for their anticancer potential, supported by evidence that diverse bioactive compounds can modulate multiple oncogenic pathways involved in tumour growth, metastasis, immune evasion and treatment resistance.* In this context, phytoceuticals are highly quantified and standardised isolates derived from plants and fungi, investigated for their therapeutic or disease‐modifying effects [34]. *These include flavonoids, terpenoids, alkaloids, carotenoids and mushroom-derived polysaccharides, which are increasingly investigated as complementary therapeutic strategies in NSCLC* [35, 36]. Among these, nobiletin, a polymethoxylated flavone found in C*itrus* species has emerged as a promising antioncogenic candidate [37, 38].

Considering the urgent need for therapies that have the potential to complement current therapies, this review is aimed at presenting a synthesis of the current evidence specifically addressing nobiletin in NSCLC. Although several reviews have examined the anticancer properties of flavonoids or citrus‐derived compounds broadly, the present review provides a focused and integrative synthesis of current evidence, specifically addressing nobiletin in NSCLC, by organising available preclinical findings and highlighting known mechanistic pathways and limitations which need addressing.

## 2. Oncogenic Pathways of NSCLC

The process of carcinogenesis involves a complex interplay of environmental influences, genetic mutations and aberrant cellular signalling among other factors that ultimately promotes cancer hallmarks such as uncontrolled cell growth, metastasis, resistance to apoptosis, immune evasion, angiogenesis and chemoresistance [15, 39]. Understanding the molecular and cellular pathways underlying these hallmarks is essential in understanding NSCLC carcinogenesis. In this section, we explore these pathways as a foundation for investigating how nobiletin may attenuate key oncogenic processes and serve as a potential therapeutic agent in NSCLC.

### 2.1. Sustained Proliferative Signalling

By nature, cancer is a disease of uncontrolled cell growth and the most fundamental trait of cancer is its ability to sustain the proliferation of malignant cells [40]. The overactivation of major pathways involved in cell growth regulation is frequently seen in many types of cancers, including NSCLC. Hyperactivation of Wingless/Integrated pathway (WNT)/*β*‐catenin, insulin‐like growth factor (IGF), Phosphatidylinositol 3‐kinase (PI3K)/protein kinase B (Akt)/mammalian target of rapamycin (mTOR) (PAM) pathways lead to downstream effects that promote proliferation, motility, survival and resistance in cancer cells [41].

One mechanism of cell proliferation is via modulation of the cell cycle [39]. The cell cycle is divided into two functional phases, S and M phases, and two preparatory phases, G_1_ and G_2_. During S phase, DNA is replicated before separation into daughter cells during M phase. Cells that remain in G_1_ for prolonged periods are called G_0_, where they remain metabolically active but are not proliferating (M et al. 2003). The advancement through these stages is tightly controlled by cyclin‐dependent kinases (CDKs) and cyclins, which are regulatory proteins that activate CDKs [42]. Therefore, overexpression of these proteins can cause rapid cell proliferation and contribute to the pathogenesis of cancer [42]. For example, Cyclin D1 mutations have been observed in up to 76% of NSCLC cancers, which drive the cell cycle forward and promote uncontrolled cell proliferation [42, 43].

### 2.2. Invasion and Metastasis

Metastasis, or the infiltration of cancer in nearby or distant tissues, constitutes the primary cause of death for > 90% of patients with cancer and is a key player in chemoradiotherapy resistance [32, 44], and thus attenuation of this process is vital in the treatment of NSCLC.

A key contributor to the metastatic potential of cancer cells is epithelial–mesenchymal transformation (EMT). EMT refers to the process of nonmotile, polarised epithelial cells becoming mobile, mesenchymal cells which, under ‘normal’ conditions, have a role in embryogenesis, inflammation, fibrosis and wound healing [45]. The major factors upregulated during EMT are the transcription factors SNAIL1/2, zinc finger e‐box binding homeobox 1 and 2 (ZEB1/2) and twist related protein 1 and 2 (TWIST 1/2). These, in turn, are regulated by interconnected signalling pathways such as rat sarcoma virus (RAS), WNT, hypoxia‐inducible factors (HIF)1/2, nuclear factor kappa‐light‐chain‐enhancer of activated B cells (NF‐*κ*B) and transforming growth factor beta (TGF‐*β*) pathway [45]. Upregulation of these pathways can suppress the expression of E‐cadherin and promote the expression of N‐cadherin and vimentin, resulting in the loss of cell adhesion and tissue integrity whilst increasing cell motility. This process may contribute to cancer cells′ ability to infiltrate surrounding tissues and metastasise to distant sites [32].

Beyond its role in motility and invasion, EMT enhances tumour aggressiveness by upregulating cancer stemness factors such as B lymphoma Mo‐MLV insertion region 1 homologue (Bmi1) and SOX family and immune checkpoint molecules like (PD‐L1), which facilitate immune evasion and multidrug resistance (MDR) [45].

### 2.3. Resistance to Apoptosis

Apoptosis is the process of programmed cell death and is a vital component of maintaining cell homeostasis. Dysregulation of this process is a key pathogenesis central to many diseases, including various types of cancers [46].

Tumour protein 53 (p53) is a tumour suppressor protein that facilitates several antioncogenic processes including cell‐cycle arrest, promoting DNA repair in damaged cells and initiating apoptosis in malignant or premalignant cells [47]. It is also involved in the activation of the proapoptotic proteins such as B‐cell lymphoma 2 (Bcl‐2) associated X protein (Bax), Bcl‐2 antagonist/killer protein (Bak) and P53 upregulated modulator of apoptosis (PUMA), which results in the degradation of antiapoptotic Bcl‐2 family members [48]. This further promotes mitochondrial outer membrane permeabilisation, release of cytochrome C and activation of caspases (caspase‐9, caspase‐3 or caspase‐7) to induce apoptosis of cancerous cells [38, 48]. Approximately 50% of cancers involve some dysregulation of p53′s signalling pathways such as upregulation of its negative regulator, mouse double minute 2 homolog (MDM2), or mutations in activating kinases such as ataxia‐telangiectasia mutated (ATM) and Checkpoint Kinase 2 (Chk2) [48].

Mutations in the gene encoding for p53 (*TP53*) were observed in over 50% of human cancers, resulting in the loss of p53′s tumour suppressive functions. The impact of p53 mutations can be seen in Li–Fraumeni syndrome, a rare inherited disorder (0.005%–0.02% worldwide) characterised by partial or full loss of function mutations in the TP53 gene [49]. Compared with noncarriers, people living with Li–Fraumeni syndrome have a 24‐fold higher chance of developing tumours [48].

### 2.4. Avoiding Immune Destruction

The immune system can sometimes play a dual role that can both inhibit and promote cancer development and progression [39, 50]. When cells from the immune system respond at the early stages of carcinogenesis, malignant cells can be destroyed to restore tissue integrity. Further, malignant cells spontaneously proliferate and overgrow the capacity of the immune response, which enables cancer cells to build a tumour microenvironment that makes it difficult to adequately eliminate cancer cells [50].

The Janus kinase (JAK)/signal transducer and activator of transcription (STAT) pathways play an important role in the immune evasion of NSCLC [33]. The JAK/STAT pathway is one of the central communication junctions that mediates numerous cellular functions. Cytokines (e.g., IL6, IL12) or growth factors (e.g., EGFR) may bind to a receptor‐associated JAK protein and induce a phosphorylation cascade to activate STATs. The STATs, in turn, translocate to the nucleus where they modulate the expression of genes involved in haematopoiesis, immunity, inflammation and apoptosis [51]. Activated STATs typically have a short lifespan; however, persistent STAT activation is a common feature in over 50% of NSCLC patients and can lead to apoptotic resistance, cell proliferation and angiogenesis [33, 52]. In tumour cells, STAT3 reduces the production of proinflammatory cytokines and chemokines resulting in accumulation of immunosuppressive cells into tumours, sustaining an immunosuppressive tumour microenvironment [52].

One such mechanism is STAT3 mediated upregulation of PD‐1. The transmembrane receptor PD‐1 is expressed primarily on activated T cells and downregulates immune response via its two ligands, PD‐L1 and PD‐L2 [53]. PD‐L1 is highly expressed in cancer cells, which negatively regulates T‐cell activity [54]. An in vitro study has shown that STAT3 promotes PD‐L1 expression in NSCLC cells [55], therefore, making the EGFR/JAK2/STAT3/PD‐L1 an attractive target in NSCLC treatment.

### 2.5. Inducing Angiogenesis

Angiogenesis is an essential mechanism that serves to maintain the supply of nutrients, oxygen and blood supply to support the tumour microenvironment. The inhibition of this process is therefore a promising strategy to limit the growth of tumours [39].

The PAM (PI3K/AKT/mTOR) pathway is one of the most significant pathways that regulate angiogenesis and cell metabolism. Aberrations involving PAM pathway occur in half of all tumours and frequently underpin the development of resistance [56]. In lung cancer, the PAM pathway is frequently overactivated due to mutations in the tumour suppressor PTEN [57]. For example, of 288 NSCLC samples the loss of PTEN was detected in 42.4% and was associated with shorter progression‐free survival [58].

Adding to its complexity, the PAM signalling network involves complex crosstalk with other signalling pathways that promote tumour heterogeneity and survival [59]. For example, the mitogen‐activated protein kinase (MEK) pathway; Wnt pathway, including NF‐*κ*B pathway, G‐protein pathway, and integrin pathway; intrinsic apoptotic pathway and p53 pathway are all closely interconnected with the PAM pathway [56]. Further, mutations in receptor tyrosine kinases (RTK), PI3K genes, PTEN and AKT have been observed to increase PAM signalling and inhibit proapoptotic proteins like BAX and BAK, and/or enhance the activity of antiapoptotic proteins [56].

The PAM signalling pathway underscores the complex, interconnected and dynamic nature of NSCLC oncogenesis and the need for novel agents that provide a synergistic and more complete blockade of PAM and its interconnected pathways in the management of NSCLC [60].

### 2.6. MDR

The emergence of drug resistance is regarded as one of the leading causes of mortality in individuals undertaking the oncology treatments [61]. The primary mechanism underlying the development of MDR is overexpression of P‐glycoprotein (P‐gp) (or ABCB1) on the membrane of MDR cancer cells [61]. P‐gp is an efflux pump that belongs to the ATP‐binding cassette (ABC) transporter family that can expel chemotherapy agents from cancer cells, decreasing their intracellular concentration and reducing the efficacy of chemotherapy [38]. The expression of P‐gp is regulated by many pathways such as PI3K, Wnt and MAPK [61]. Consequently, many drugs have been trialled to inhibit P‐gp activity; however, they have been marred by adverse effects, poor pharmacokinetic profiles and inferior clinical outcomes in a range of cancer models [61–63].

A second member of the ABC transporter family, multidrug resistance‐associated protein 1 (MRP1) (or ABCC1), is commonly overexpressed in NSCLC and promotes the efflux of many chemotherapeutic drugs in tumour cells, reducing their cytotoxicity [64]. In a meta‐analysis, MRP1 was significantly enhanced (OR, 5.54; 95% CI, 3.69–8.32; *p* < 0.0001) in the cancerous tissue of NSCLC compared with nontumour pulmonary tissue adjacent to the affected tissues [64]. MRP1 expression is also associated with poorer outcomes in NSCLC patients [65]. Accordingly, researchers have turned their attention to natural products as potential new generation MDR inhibitors [66, 67].

## 3. Therapeutic Potential of Plant‐Based Medicines Against NSCLC

Although the earliest records of plant‐based medicines can be traced as far back as 2900 BCE to the Ebers Papyrus of ancient Egypt, the use of plant‐based medicine systems predates written history [68, 69]. However, it was not until Friedrich Wilhelm Adam Serturner isolated morphine from the opium poppy in 1805 that the foundations were laid for the methodical study of ‘modern’ plant‐based isolates [70, 71]. Today, much of the developing world relies on traditional medicine systems, and it is estimated that 80% of the global population relies on traditional medicine as their primary source of healthcare [70]. Similarly, the use of complementary and alternative medicines (CAMs) has been embraced in the developed world, reflecting the social shift towards a more natural, holistic approach to healthcare [72]. Australia has one of the highest rates of CAM use among developed countries, with up to 50% of individuals reporting CAM use within the past year and industry sales surpassing AUS$5.69 billion in 2020 [73].

Phytochemical groups include polyphenols, terpenes, sulphurous compounds, nitrogenous compounds, phthalides, phytosterols and carotenoids [35]. A summary of the main classes of phytochemicals is depicted in Table 1. Polyphenols, a secondary plant metabolites, with more than 10,000 identified compounds, play a key role in plant growth, reproduction, colour and defence mechanisms [79]. They are most commonly found in fruits, wine, tea, coffee and beer, and in lower quantities in vegetables, legumes and grains [80]. Polyphenols are characterised by the presence of at least one phenol ring and one or more hydroxyl (‐OH) substituents. There are two basic categories of polyphenols: flavonoids and nonflavonoids (lignans, stilbenes, phenolic acids and nonphenolic metabolites) which range from simple, single aromatic ring structures to large, complex molecules such as secoisolariciresinol [74, 81].

**Table 1:** Classification of some of the main classes of phytoceuticals based on chemical structure, sources and examples.

Class	Chemical structure	Sources	Examples	Author(s)
Polyphenols	Phenol ring with ≥ 1 hydroxyl group.	Oranges, wine, tea, coffee.	Resveratrol, curcumin, nobiletin	[74],[75]
Terpenoids	Five‐carbon isoprene backbone with various functional groups.	Grapes, flowers, wood.	Propolis, *Ginkgo biloba*, cannabinoids	([66, 67], [76])
Carotenoids	40‐carbon backbone with a central 22C chain and two 9C acyclic or cyclic end groups.	Tomatoes, carrot, *Macula lutea.*	Carotene, lutein, lycopene	([77], [66, 67])
Sulphur‐containing compounds	Characterised by the presence of sulphur in their structure.	Onion, garlic, cruciferous vegetable.	Allicin, sulforaphane	[78]
Nitrogenous compounds	Characterised by the presence of nitrogen in their structure.	Legumes, herbs, some roots and rhizomes.	Berberine, caffeine	[35]

Terpenoids include compounds such as menthol, citronellal and camphor and are found in eucalyptus (*Eucalyptus* L.), lime (*Citrus latifolia* L.) and orange (*Citrus sinensis* L.) [66, 67]. All terpenoids comprise five‐carbon isoprene units, and based on the number of these units, they can be further classified as monoterpenes, sesquiterpenes, diterpenes, sesterterpenes, triterpenes, tetraterpenes and polyterpenes [76]. These compounds have been studied for their antibiotic, antiallergic, antioxidant and antioncogenic properties in vitro and in animal models [82].

Carotenoids are bright yellow, red and orange coloured pigments found in fruits and vegetables such as tomatoes, peaches, mango, sweet potato, carrot and pumpkin [66, 67]. Carotenoids consist of a 40‐carbon backbone, with a central 22‐carbon chain flanked by two 9‐carbon terminal units, which may be either acyclic or cyclized at one or both ends and can bear oxygen‐containing functional groups [77]. These compounds are potent antioxidants and have been studied for their role in attenuating cardiovascular disease, Type 2 diabetes, skin and eye disease, bone disease and a range of cancers, including lung cancer [77].

Sulphur compounds are found in cruciferous vegetables including broccoli (*Brassica oleracea*), cauliflower (*B*. *oleracea*), onion (*Allium cepa*) and garlic (*Allium sativa*) and are associated with inhibiting the inflammatory response, reducing free radicals and promoting immune functions [78]. Nitrogen containing compounds include the alkaloids, cyanogenic glucosides and nonprotein amino acids [35]. Alkaloids are the most extensive class of nitrogenous compounds and have been extensively studied for their potent role in attenuating cancer, depression, malaria and hypertension [83]. Phytosterols are plant sterols and stanols, which have benefits in prostate health, hair growth, LDL cholesterol lowering effect and antioxidant activity [66, 67].

Flavonoids, which make up approximately two‐thirds of the polyphenols, have the C6–C3–C6 general structural backbone, in which the two C6 units are phenolic. Due to the hydroxylation and methylation patterns of the chromane ring, flavonoids can be further divided into different subgroups based on their structure such as anthocyanins, flavan‐3‐ols, flavones, flavanones and flavonols [84]. Dietary consumption of flavonoids has been shown to have anti‐inflammatory, antioxidant, antibacterial, antiviral and anticancer activities [85]. A recent meta‐analysis from Liu et al. proposed that 200 mg of dietary flavonoid consumption may reduce the risk of all‐cause mortality by 18% [80, 86]. Therefore, it is unsurprising that there is a plethora of research investigating the therapeutic potential of these compounds in treating many chronic diseases [87–89], including NSCLC [90–93].

## 4. Nobiletin

Citrus peels have long been utilised for their therapeutic properties in traditional medicine systems. *Citri reticulatae pericarpium* is derived from the ripe peel of the *Citrus reticulata* and has long been used in traditional Chinese medicine (or, Chen Pi/??) to manage nausea, vomiting, indigestion, diarrhoea, and cough, and to regulate “Qi” [94, 95]. To date, over 140 chemical compounds have been isolated from *Citri reticulatae pericarpium*, with flavonoids being the main bioactive ingredient [95].

**Figure 2:**
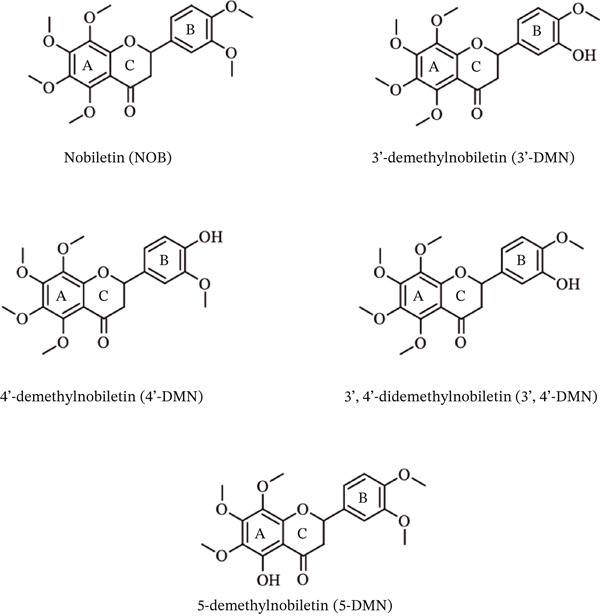
Chemical structure of nobiletin compared with some of its main metabolites where after oral administration, nobiletin is rapidly transformed to its demethylated or di‐demethylated metabolites like 3 ^′^DMN, 4 ^′^‐DMN and 3,4 ^′^‐DMN.

Nobiletin (5,6,7,8,3,4‐hexamethoxyflavone), whose chemical structure is depicted in Figure 2, is a bitter tasting polymethoxylated flavonoid, derived from the peel of *Citrus* fruits. It is found in high concentrations in *Citrus depressa* (Shiikuwasha), *C. reticulata* (Ponkan mandarin), *Citrus kinokuni* (Mukaku kishu), and *Citrus tumida* (Fukure mikan) [75].

Numerous studies have demonstrated that nobiletin has a broad range of potential therapeutic applications, which are outlined in Table 2. Additionally, nobiletin has shown anticancer activity in a range of cancers including, but not limited to, cancers of the oral cavity [102], breast [103–105], gastrointestinal tract [106], ovary [107] and prostate [108]. Therefore, it is also plausible that there may be therapeutic potential of nobiletin in the management and treatment of NSCLC.

**Table 2:** A summary of the studies of nobiletin in a number of different disease states.

Study type	Disease	Key outcome(s)	Author
In vivo	Alzheimer′s disease (AD)	NOB significantly alleviated *β*‐amyloid burden, reduced inflammatory cytokine expression, and appeared to improve cognitive function in amyloid precursor protein/presenilin 1 (APP/PS1) mouse model of AD.	[96]
*In vivo*	Pulmonary arterial hypertension	Intragastric administration of nobiletin attenuated monocrotaline‐induced pulmonary artery hypertension, pulmonary vascular remodelling and right ventricular hypertrophy in Sprague–Dawley (SD) rats by inhibition of inflammatory markers via downregulation of PI3K/Akt/STAT3 signalling.	[97]
In vitro and in vivo	Atherosclerosis	Nobiletin inhibited lipid uptake in RAW264.7 macrophage cells, thereby preventing foam cell formation essential to atherosclerosis parthenogenesis. Nobiletin also reduced total cholesterol and triglyceride levels and reduced atherosclerotic plaque production and lipid accumulation in aortic valves of ApoE−/− mice.	[98]
In vivo	Type 2 diabetes mellitus	Nobiletin treatment in obese diabetic ob/ob mice significantly reduced plasma glucose levels and insulin sensitivity, increased adiponectin expression and decreased inflammatory adipokines.	[99]
In vivo	Obesity	Nobiletin treatment in high‐fat diet‐induced obese mice significantly reduced body weight gain, white adipose tissue mass, plasma triglycerides and improved glucose tolerance and insulin sensitivity.	[100]
In vivo	Circadian regulation	Nobiletin in mice induced time‐of‐day‐dependent changes in peripheral circadian clocks, advancing or amplifying clock gene rhythms whilst acutely stimulating corticosterone and adrenaline secretion in a novel adrenal hormone–mediated mechanism for circadian modulation.	[101]

Nobiletin is a white–yellow crystalline powder sourced by solvent extraction, microwave extraction, ultrasonic extraction, enzyme extraction and Soxhlet extraction method [37, 109]. Further, nobiletin can be produced by total chemical synthesis methods such as the resorcinol method and the flavonoid oxidation method, or by using a semisynthetic approach to chemically modify pre‐existing flavonoids [109].

**Figure 3:**
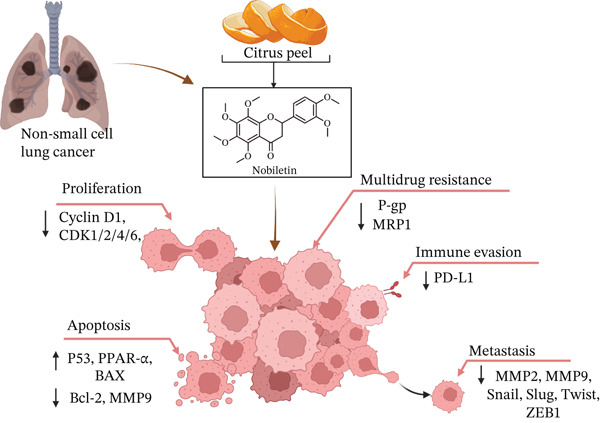
Nobiletin, a polymethoxylated flavonoid isolated from citrus peels, may impact a number of key cancer hallmarks involved in nonsmall cell lung cancer. Nobiletin has shown promising in vitro and in vivo inhibition of oncogenic signalling pathways that regulate cell cycle progression, proliferation and metastasis. It further enhances proapoptotic signalling, downregulates survival pathways, and mitigates chemoresistance by modulating multidrug resistance proteins, highlighting its potential as a therapeutic agent in nonsmall cell lung cancer (image created using https://BioRender.com).

Due to the presence of six methoxyl groups located at Positions 5, 6, 7 and 8 on the A ring, as well as Positions 3 ^′^ and 4 ^′^ on the B ring (Figure 2), nobiletin is highly lipophilic and rapidly absorbed [110]. Following oral administration, nobiletin is digested in the upper gastrointestinal tract and absorbed in the jejunoileal [109] and accumulates in the brain approximately three times higher than in plasma when administered to male Sprague–Dawley rats following oral administration of nobiletin at 50 mg/kg [111]. Nobiletin undergoes Phase I metabolisation by demethylation in the liver through CYP 450 enzymes. The primary metabolites (Figure 3) are 3 ^′^‐demethylnobiletin (3 ^′^‐DMN), 4 ^′^‐demethylnobiletin (4 ^′^‐DMN), and 3 ^′^,4 ^′^‐didemethylnobiletin (3 ^′^,4 ^′^‐DMN), 5‐demethylnobiletin (5‐DMN) [110, 112, 113]. In a study by Wu et al. levels of nobiletin were approximately 20‐fold lower than total level of metabolites in the colonic mucosa, suggesting it is rapidly metabolised into its active constituents [112, 113]. The anticancer potential of these molecules is discussed in detail below. Phase II metabolism takes place in the small intestine where it undergoes sulfation and glucuronidation reactions [110]. Following metabolism, nobiletin is distributed throughout the body with highest concentrations found within the small intestines and hepatic tissues, followed by gastric and adipose tissues. Urinary and faecal excretions of nobiletin accounted for 7% and 8%, respectively, of the total administered dose [109].

Drugs can be classified based on the Biopharmaceutics Classification System (BCS): high solubility–high permeability (Class I), low solubility‐high permeability (Class II), high solubility–low permeability (Class III), and low solubility–low permeability (Class IV) [114]. Nobiletin has been classified as a BSC Class IV compound due to its low water solubility (16.2 *μ*g/mL) and very low bioavailability (0.85%) in rats [115]. This, in part, may be attributed to the flavone backbone, methoxylation patterns of the hydroxyl groups, and the significant first‐pass and Phase II metabolism of nobiletin [116, 117].

### 4.1. Toxicity of Nobiletin

The toxicity of nobiletin has not been studied extensively in vitro and in vivo, and further studies on a range of cell types and tissues are required [38]. However, nobiletin is derived from natural sources and based on current in vitro studies, and it appears to have a low toxicological profile. For example, nobiletin (10, 20 and 40 *μ*g/mL) did not affect colony formation of human umbilical vein endothelial (EVC304) cells whilst inhibiting colony formation of adenocarcinomic human alveolar basal epithelial A549 cells, suggesting nobiletin may function selectively between healthy, noncancerous cells and NSCLC cells [118]. Further, several in vivo studies in mice did not demonstrate loss of body weight, a traditional marker of toxicity [119–121]. However, it should be noted that often phytoceuticals are considered to be nontoxic because of their low bioavailability; however, toxicity may be a concern when systemic bioavailability is markedly increased [122].

## 5. Therapeutic Potential of Nobiletin in NSCLC

The existing evidence for nobiletin′s anticancer potential, structured around the hallmarks of cancer, will be discussed in Section 5 of this review [39]. The findings of preclinical NSCLC studies are summarised in the following subsections and depicted in Table 3. Collectively, these findings highlight that nobiletin exerts an overlapping biological activity across different NSCLC models, which further necessitates the need for a systemic, hypothesis‐driven approach to screen the most effective and pertinent therapeutic intervention in the disease management.

**Table 3:** Summary of preclinical studies investigating the anticancer effects of nobiletin in NSCLC models.

Study type	Concentration/dosage	Primary findings	Significance	Hallmark affected	Reference
In vitro	2.5 *μ*M	↑ NKD1, AXIN2, WIF1, DKK1	Nobiletin treatment is negatively correlated with EMT and cancer stemness in H460 and H549 cells.	Invasion and metastasis.	[123]
		↓ *β*–catenin and downstream genes WNT6 and JUN			
In vitro	0, 10, 20 and 40 *μ*M	↑ PPAR‐*α* signalling	Nobiletin inhibits the proliferation and colony forming ability of NSCLC in a dose‐dependent manner in A549 cells.	Proliferation and invasion and metastasis.	[124]
		↑ p53			
		↓ MMP9			
In vitro and in vivo	10 *μ*g/mL (24.9 *μ*M), 20 *μ*g/mL (48.7 *μ*M), and 40 *μ*g/mL (99.4 *μ*M)	↓ Bcl‐2	Nobiletin suppressed proliferation of A549 cells in vitro and inhibits tumour growth in nude mice.	Apoptosis.	[118]
		↑ Bax protein			
		↑ p53			
	100 mg/kg, 200 mg/kg and 300 mg/kg (oral once daily administration for 15 days)				
In vitro and in vivo	20, 40, and 80 *μ*M	↓ CDK4, CDK2, cyclin D1	Nobiletin has a synergistic relationship with paclitaxel through G1 cell cycle arrest in A549 and H460 cells. A549 tumour growth was supressed in vivo.	Proliferation.	[121]
	600 *μ*g in 300 *μ*L (oral alternate day administration for 30 days				
		↓ Cleaved caspase‐3 and cleaved PARP			
		↑ Bcl‐2.			
In vitro and in vivo	10, 20, 30, 40 and 50 *μ*M	↑ Bax protein, p21Cip1/Waf1, cleaved caspase‐3 and cleaved PARP	Oral administration of NBT significantly inhibited NKK induced NSCLC model in mice.	Proliferation and apoptosis.	[120]
	0.05% (*w*/*w*) in AIN‐76A diets (orally administered via feed)	↓ cyclin D1, CDK1, CDK6			
In vitro and in viv*o*	0, 20, 40, 60, 80, 100 and 120 *μ*M	↓ TGF *β*1/Smad3 signalling	Inhibition of EMT, migration, invasion and adhesion in vitro in A549 and H1299 cells. Reduced tumour growth and metastasis in A549 inoculated mice.	Invasion and metastasis.	[125]
	20 and 40 mg/kg (oral once daily administration)				
	↓ MMP2, MMP9, p‐Src, p‐FAK, p‐paxillin, snail, slug, twist and ZEB1				
In vitro	0.5, 1.5, 4.5 and 9 *μ*M	↑ABCB1 ATPase activity	Nobiletin significantly increased chemosensitivity of A549 (paclitaxel‐resistant) cancer cells to paclitaxel by acting as a competitive inhibitor of P‐gp.	Multidrug resistance.	[126]
		↓ AKT/ERK/Nrf2 pathway			
In vitro	100 and 200 *μ*M	↓ EGFR/JAK2/STAT3 signalling and PD‐L1 expression	Nobiletin suppresses immune evasion in a p53‐ independent mechanism via downregulation of PD‐L1 expression in A549, H292 and H460 cells.	Immune evasion.	[127]
		↓ MDM2 and expression of p53			
In vitro and in vivo	50 *μ*M	↓ Akt/GSK3*β*/*β*–catenin/MYCN signalling pathway	Coadministration of nobiletin with doxorubicin induced apoptosis of doxorubicin‐resistant A549 cells and reduced tumour volume by 84.15%.	Multidrug resistance.	[119]
	40mg/kg (route of administration not specified)				
	↓ MRP1 and MYCN protein expression				
In vitro and in vivo	9 *μ*M	↓ P‐gp	Nobiletin reduced cancer cell colony formation and increased chemosensitivity of A549 cells to paclitaxel.	Multidrug resistance.	[128]
		↓ Nrf2/PI3K/AKT pathways.			
	25 and 50 mg/kg (intraperitoneal injection)				

### 5.1. Inhibition of Proliferation and Induction of Apoptosis

Nobiletin is proposed to have promising antiproliferative and proapoptotic activity in both in vitro and in vivo NSCLC models. Treatment of human large cell NSCLC (H460 cells and A549 cells) with nobiletin significantly reduced colony formation via upregulation of the proapoptotic proteins cleaved PARP and cleaved caspase‐3 [123]. Yao et al. [124] further showed that nobiletin dose‐dependently increased the mRNA and protein expression of P53, whilst upregulating PPAR‐*α* and downregulating MMP9 in A549 cells. These molecular changes also significantly reduced colony formation when cells were treated with 20 and 40 *μ*M of nobiletin [124].

The cell cycle arrest and apoptosis in the G2/M phase in A549 was induced after treatment with nobiletin (99.5 *μ*M). Protein expression analysis showed that nobiletin treatment increased Bax and p53 expression whilst decreasing antiapoptotic Bcl‐2 levels [118]. Bcl‐2 is commonly overexpressed in NSCLC, inducing cancer growth, angiogenesis, resistance to chemotherapeutics [129]. The attenuation of this pathway by nobiletin may be a promising mechanism of nobiletin′s anticancer effect. Interestingly, Uesato et al. [121], observed an increase in Bcl‐2 and decreased apoptotic cleaved caspase‐3 and cleaved PARP levels when nobiletin was administered with paclitaxel and carboplatin. Instead, the proportion of cells in G0 arrest increased to inhibit proliferation of A549 and H460 NSCLC cell lines [121]. Despite these promising effects, nobiletin only modestly induced G0 cell cycle arrest in human nonsmall cell lung carcinoma H1299 cells and had limited impact on cell cycle progression in H460 cell lines in a later study from Sun et al. [120] [120]. These results suggest that although nobiletin has the potential to influence cell proliferation and apoptosis, its ability to disrupt the cell cycle machinery may be suboptimal in certain NSCLC cell lines and requires further research to fully elucidate its antiproliferative effect.

Studies on nobiletin treatments in vivo reduced average tumour volume by 40.5%, compared with the positive control group (0.25 ± 0.02  mm^3^ vs. 0.42 ± 0.01 mm^3^) in a mice model of 4‐(methylnitrosamino)‐1‐(3‐pyridyl)‐1‐butanone (NNK) induced lung cancer [120]. Similarly, Luo et al. [118] found that tumour weight in nude mice heterotopically inoculated with A549 cells was significantly reduced in the nobiletin treated group compared with the cyclophosphamide treated group (Luo et al. [118]).

The antiproliferative and proapoptotic effects of nobiletin have been primarily demonstrated in a limited number of cell lines, most notably A549, H460 and H1299, which differ in their TP53 status and oncogenic backgrounds. Several studies report p53 activation and upregulation of downstream apoptotic markers such as Bax, cleaved caspase‐3 and PARP following nobiletin treatment, suggesting a p53‐dependent component to its anticancer activity [118, 124]. However, inhibitory effects on proliferation and survival have also been observed in p53‐null models such as H1299 cells, indicating that p53‐independent mechanisms, potentially involving PI3K/Akt, Wnt/*β*‐catenin or cell‐cycle regulatory pathways, may also contribute [120, 123]. To date, few studies have examined nobiletin in NSCLC models stratified by clinically relevant driver mutations, such as EGFR or KRAS, limiting conclusions regarding genotype‐specific responsiveness. Consequently, further studies using genetically defined NSCLC systems are required to clarify how nobiletin′s antiproliferative and proapoptotic effects translate across common molecular subtypes of the disease.

### 5.2. Suppression of Invasion and Metastasis

Nobiletin has been shown to play a role in suppressing EMT in in vitro models. Nobiletin suppressed transcription factors SNAIL, Slug, ZEB1 and TWIST to restore epithelial related characteristics in A549 and human adenocarcinoma H1299 cells [125]. Further, nobiletin inhibited expression of matrix metalloproteinase (MMP) 2 and MMP‐9 [125], which are responsible for breakdown of the extracellular matrix to promote cell migration, angiogenesis and proliferation [130]. Nobiletin upregulated inhibitors of Wnt/*β*‐catenin such as naked cuticle 1 (NKD1), AXIS inhibition protein 1/2 (AXIN2), Wingless/Integrated pathway inhibitory factor 1 (WIF1) and Dickkopf‐1 (DKK1) and suppressed positive regulators wingless‐type MMTV integration site family 6 (WNT6) and JUN to reduce cancer cell stemness and migration in A549 and H460 cells [123].

Nude mice injected with A549 cells were intravascularly given once daily doses of oral nobiletin of 20 and 40 mg/kg and showed significantly reduced metastatic nodules in the lung (43 ± 11 and 29 ± 9 nodules, respectively) compared with the cyclophosphamide control group (65 ± 15 nodules, *p* < 0.05) after 42 days of treatment, highlighting its potent antimetastatic potential [125].

Current evidence suggests that nobiletin primarily suppresses tumour cell intrinsic metastatic mechanisms in NSCLC, including EMT‐associated transcription factors, matrix metalloproteinases and Wnt/*β*‐catenin signalling. Whether nobiletin also modulates tumour–stroma interactions, immune components of the lung microenvironment, premetastatic niche formation, or circulating tumour cell dynamics remains unclear and warrants further investigation

### 5.3. Chemosensitivity, MDR and Immune Evasion

One of the challenging hallmarks of NSCLC is the development of chemoresistance, which severely limits the effectiveness of standard chemotherapy agents. Nobiletin has been shown to sensitise paclitaxel resistant A549/T cells by inhibiting P‐gp (or, ABCB1). By acting as a competitive inhibitor, nobiletin reduces the efflux activity of P‐gp and enhances the intracellular accumulation of paclitaxel to improve its cytotoxic effect [126]. In addition to its effect on P‐gp, nobiletin downregulates several signalling pathways that promote the MDR phenotype such as nuclear factor erythroid 2‐related factor 2 (Nrf2), PI3K/Akt and extracellular signal‐regulated kinase (ERK) [128]. The joint suppression of these signalling pathways and inhibition of P‐gp demonstrates the potential of nobiletin as a chemo‐sensitising agent in MDR NSCLC. Further, nobiletin treatments at concentrations between 0.5 and 9 *μ*M in A549 paclitaxel resistant cells decreased the IC50 of paclitaxel by 60.1% and 98%, respectively [126]. Complete colony formation inhibition was achieved when low‐dose paclitaxel was combined with nobiletin, highlighting their potential synergistic relationship [126].

In doxorubicin (Adriamycin) resistant A549 cells (A549/ADR), nobiletin treatment decreased MRP1/ABCC1 via suppression of the Akt/GSK3*β*/*β*–catenin/MYCN signalling pathway. This enhanced the intracellular accumulation of doxorubicin and restored drug sensitivity [119]. In female BALB/c nude mice subcutaneously inoculated with A549/ADR cells, tumour volume with doxorubicin was 22.93% (± 18.42) lower compared with control, whereas tumour volume decreased by 42.61% (± 5.73) in the treatment group. Furthermore, coadministration of nobiletin with doxorubicin reduced tumour volume by 84.15% (± 11.54) with no observed systemic toxicity, highlighting the potential use of nobiletin as an adjunct to traditional chemotherapy agents [119]. In a study from Uesato et al. [121], concomitant nobiletin/paclitaxel/carboplatin did not significantly reduce cell tumour volume, but had a more favourable toxicity profile compared with traditional paclitaxel and carboplatin chemotherapy in female BALB/c‐nu.nu nude mice subcutaneously inoculated with A549 cells [121]. Furthermore, in nude mice subcutaneously injected with A549 paclitaxel resistant tumour cells, nobiletin enhanced paclitaxel accumulation in the tumour by almost threefold compared with paclitaxel alone with no evidence of weight loss in all mice [128].

Although there is less known about nobiletin′s role in attenuating immune evasion, tumour immune escape is a common process in the tumourigenesis of NSCLC and a major challenge in NSCLC treatment. Nobiletin has been shown to suppress PD‐L1 levels by more than 50% in A549, H292 and H460 cells via suppression of activation of the EGFR/JAK2/STAT3 signalling cascade [127]. Furthermore, although current studies demonstrate that nobiletin enhances chemosensitivity in multidrug‐resistant NSCLC models, including reductions in IC50 values and tumour burden alongside suppression of P‐gp and MRP1, these findings primarily support functional chemosensitisation rather than definitive reversal of MDR. Confirmation of transporter‐specific MDR reversal will require future studies employing genetic knockdown or knockout of efflux transporters, use of nonsubstrate cytotoxic agents and functional efflux assays to distinguish direct transporter inhibition from general cytotoxic additivity.

### 5.4. Anticancer Activity of Nobiletin Metabolites

Biotransformation plays a critical role in determining the biological activity of naturally sourced compounds. Studies have shown that the major metabolites of nobiletin have higher biological activity and may be effective in reducing inflammation [112, 113, 131] and may be antioncogenic [112, 113, 120, 128, 132]. As such, emerging research has focused on the anticancer activity of nobiletin’s metabolites in NSCLC.

An in vitro study using H1299 cells demonstrated that mono‐demethylated metabolites of nobiletin increased cell cycle arrest and the expression of proapoptotic proteins caspase‐3 and poly (ADP‐ribose) polymerase (PARP) [133]. Similarly, these metabolites were shown to have a stronger antiproliferative and proapoptotic activity in H1299 and H460 cells compared with intact nobiletin [134]. A subsequent study by Sun et al. [120] demonstrated greater anticancer activity of nobiletin metabolites than nobiletin itself. In H460 cells, the IC50 of 4 ^′^‐DMN and 3 ^′^,4 ^′^‐DMN were approximately 2.9‐fold and 5.3‐fold lower than that of nobiletin alone, respectively. This led to upregulation of proapoptotic Bax, cleaved caspase‐3 and cleaved PARP, and downregulated cell‐cycle drivers cyclin D1, CDK4 and CDK6 [120]. These findings underscore the therapeutic potential of nobiletin’s demethylated metabolites in the treatment of NSCLC and highlight the need for future in vivo studies.

## 6. Discussion and Future Directions

The studies discussed in the present review, summarized in Figure 3, highlight the promising therapeutic potential of nobiletin against NSCLC. Despite this, its clinical application, similar to that of many other natural products, is still hampered by its poor bioavailability, resulting from poor solubility and poor permeability patterns [135, 136].

The therapeutic potential of natural compounds continues to be one of the topics with high research interest, a new body of research regarding nanoparticle‐based drug delivery systems (particles sized 1–100 nm) is rapidly developing. The use of nanotechnology applications with natural products can improve solubility, bioavailability, specificity and avoidance of first pass metabolism [137] via products such as polymer nanoparticles, solid lipid nanoparticles, liquid crystalline nanoparticles, liposomes, micelles, dendrimers and others [137, 138]. The use of these advanced drug delivery systems holds the chance to fully exploit the untapped potential of many plant‐derived molecules with promising biological activity whose further development is hampered by poor bioavailability [139].

Another suitable strategy to improve nobiletin′s bioavailability involves applying innovative formulation strategies. When administered as an insoluble suspension, nobiletin was shown to have lower plasma concentration and slower absorption when compared with nobiletin solubilised in corn oil [140]. These findings suggest that presolubilisation can improve the pharmacokinetic profile of nobiletin and support the rationale to investigate novel formulation strategies to enhance nobiletin′s solubility and, therefore, improve its bioavailability. For example, Onoue et al. [115] investigated the use of wet milling techniques to produce an amorphous solid dispersion of nobiletin. This approach resulted in a 13‐fold improvement in the oral bioavailability of nobiletin, improving the absolute bioavailability from 0.85% to approximately 11% [115]. Although this is a substantial improvement, the absolute bioavailability remains low and demonstrates the need for further optimisation.

Many techniques have sound potential to improve the oral bioavailability of nobiletin; however, a definitive strategy is still inconclusive, and novel delivery approaches have been investigated. For example, an ionic liquid optimised formulation of nobiletin delivered via a transdermal patch produced a bioavailability of 9.04% in rats [116]. Zhang et al. [141] developed an inhaled nano formulation of fingolimod, nobiletin and poly lactic‐co‐glycolic acid nanoparticles with improved biocompatibility of nobiletin for use in acute lung injury [141].

Additionally, although nobiletin has generally shown a favourable safety profile in preclinical models, including minimal effects on body weight and limited toxicity towards nonmalignant cells, comprehensive assessment of organ‐specific toxicity and long‐term exposure effects remains lacking. Moreover, given nobiletin′s ability to modulate major signalling pathways and efflux transporters such as P‐gp and MRP1, the potential for pharmacokinetic or pharmacodynamic interactions with standard NSCLC therapies cannot yet be excluded. Dedicated toxicological and drug–drug interaction studies will therefore be essential to define the safety of nobiletin‐based combination strategies.

Despite the promising advances, there is no single successful strategy, and the development of an optimal delivery system for nobiletin remains ongoing. Additionally, no human clinical trials have been conducted to assess nobiletin′s efficacy in NSCLC, nor other cancers. Given the promising in vitro and in vivo preclinical evidence, clinical studies exploring nobiletin as a potential adjunct to conventional NSCLC therapies are warranted.

## 7. Conclusion

NSCLC remains a leading cause of cancer‐related mortality worldwide, with treatment efficacy hindered by diagnostic delays, therapeutic resistance and high recurrence rates, highlighting the urgent need for novel interventions. Nobiletin, a polymethoxylated flavonoid, has demonstrated anticancer potential across various malignancies and has emerged as a candidate of interest in NSCLC. Although preclinical evidence suggests promising antitumour activity, its clinical translation is currently limited by poor bioavailability. Future research should prioritise systematic evaluation of nobiletin across distinct NSCLC subtypes to determine context‐dependent efficacy and pathway specificity. In parallel, rational combination strategies with standard chemotherapeutic, targeted, or immunotherapeutic agents should be explored to define synergistic interactions, optimise dosing and assess potential roles in overcoming therapy resistance. Taken together, nobiletin represents a promising phytoceutical that warrants further investigation to clarify its potential role in supporting existing therapeutic strategies for NSCLC.

## Author Contributions

**A.H-M.:** writing, manuscript concept and design, draft preparation, review and editing, supervision and conceptualization. **J.B.:** manuscript concept and design, image concept, design and creation and writing—original draft preparation. **S.Y.:** review. **K.R.P.:** review. **N.N.:** writing—review and editing. **D.C.:** review. **H.C.:** writing—review and editing. **P.P.:** chemical structure concept. **B.G.G.O.:** writing—review and editing. **F.W.:** review. **K.D.:** conceptualisation, supervision and writing—review and editing. **G.D.R.:** writing—review and editing, supervision and conceptualisation. **A.H-M. and J.B.** have contributed to the work equally and should be regarded as co‐first authors.

## Funding

Open access publishing was facilitated by the University of Technology Sydney, as part of the Wiley ‐ University of Technology Sydney agreement via the Council of Australasian University Librarians.

## Conflicts of Interest

The authors declare no conflicts of interest.

## Data Availability

Not applicable—the present manuscript is a review and does not include any generated data.
